# Clinical and demographic characteristics of adenomatoid odontogenic tumors: analysis of 116 new cases from a single center

**DOI:** 10.1016/j.bjorl.2020.06.004

**Published:** 2020-07-21

**Authors:** Bogahawatte Samarakoon Mudiyanselage Samadarani Siriwardena, Muthuranwelli Nawaragoda Gedara Pushpakumara Udagama, Tennakoon Mudiyanselage Priyanka Bandara Tennakoon, Demin Achchi Athukoralalage Dona Wimukthi Athukorala, Primali Rukmal Jayasooriya, Wanninayake Mudiyanselage Tilakaratne

**Affiliations:** a University of Peradeniya, Faculty of Dental Sciences, Kandy, Sri Lanka; b Ministry of Health, Kandy, Sri Lanka; c University of Peradeniya, Faculty of Allied Health Sciences, Sri Lanka; d University of Malaya, Faculty of Dentistry, Department of Oral and Maxillofacial Clinical Sciences, Malaya, Malaysia

**Keywords:** Adenomatoid odontogenic tumors, AOT, Relative frequency, Odontogenic tumors, Demography

## Abstract

**Introduction:**

The adenomatoid odontogenic tumor is a relatively uncommon odontogenic neoplasm representing about 4.7% of all odontogenic tumors.

**Objective:**

The aim of this study was to determine the demographic and clinical profile of the adenomatoid odontogenic tumors in a Sri Lankan population.

**Methods:**

Data gathered from the cases received for a period of 38 years from the Department of Oral Pathology, Faculty of Dental Sciences, University of Peradeniya. Request forms, biopsy reports and electronic data base of the department were used to obtain relevant information. Demographic data including age, gender and location of the tumor were included in the analysis.

**Results:**

Out of 116 cases of adenomatoid odontogenic tumor, the mean age was 21.02 ± 11.24. It occurs more fre quently in the second decade of life, more prevalent in females, most often associated with the maxilla, predominantly affecting anterior jaw bones and presenting mostly in the right side of the jaw bone. The results from the present study showed the statistically significant relationship with site of occurrence (maxilla/mandible) and age (*p* < 0.005). Further, depending on whether it occurs in anterior/mid/posterior site also showed a significant relationship with age (*p* ≤ 0.001). However, side of occurrence, left or right or site of occurrence, showed no statistically significance with age (*p* > 0.05).

**Conclusion:**

Adenomatoid odontogenic tumor occurs more frequently in the second decade of life with a significant female predominance and the commonest site is anterior maxilla. This study revealed few differences on demographic and clinical presentations of adenomatoid odontogenic tumor from some regions of the world.

## Introduction

Odontogenic Tumors (OT) are uncommon lesions that originate from epithelial, ectomesenchymal and/or mesenchymal tissues of the tooth-forming apparatus. It constitutes a heterogeneous group of lesions with diverse biological, clinical, and histopathological features ranging from benign lesions to malignant tumors.^1^, ^2^ The classification of odontogenic tumors is essentially based on these interactions between odontogenic ectomesenchyme and epithelium.^3^ The literature indicates that odontogenic tumors show a geographic variation in their distribution and frequency.^3^, ^4^, ^5^, ^6^, ^7^

The Adenomatoid Odontogenic Tumor (AOT) is classified under epithelial odontogenic tumors due to histomorphologic resemblance of the components of the dental organ. AOT is a benign (hamartomatous) odontogenic lesion that has been regarded either as a non-invasive and non-aggressive neoplasm with slow but progressive growth. AOT is generally considered to be an uncommon tumor.^8^

The search for the first identifiable AOT case is challenging because many names have been used for this entity. Some early cases were grouped together with other superficially similar tumors and this was further complicated as photomicrographic documentation was not available in that era. AOT was first described by Dreibaldt in 1907, as “pseudo-adenoameloblastoma”. Harbitz et al. published in 1915 as a “cystic adamantoma”.^9^

The first series of AOT were reported by Stafne in 1948 under the title “epithelial tumors associated with developmental cysts of the maxilla”.^10^ Bernier and Tiecke published an article that was the first case to use the name “adeno-ameloblastoma”.^11^

Terminology used for AOT varies according to the literature. Miles from England reported it as “Cystic complex composite odontome”.^12^ Further, Oehlers from Singapore as “an unusual pleomorphic adenoma-like tumor in the wall of a dentigerous cyst”,^13^ Lucus from London as “tumor of enamel organ epithelium” (Lucus, 1957), Japanese author as “adenomatoid ameloblastoma”^14^ and Smith from United States as “adenomatoid odontoma”^15^ were the other nomenclature used for the same entity. There were AOT cases documented as “adenoameloblastoma”, “ameloblastic adenomatoid tumor”, “adamantinoma”, “epithelioma adamantinum” and “teratomatous odontoma”. Finally, in 1969 Philipsen and Birn proposed the widely accepted name “adenomatoid odontogenic tumor”.^16^

Like all other odontogenic tumors, the specific stimulus that triggers proliferation of the progenitor cells of AOT is unknown. AOT accounts for approximately 3%–7% odontogenic tumors and is the fourth most frequent tumor among OTs.^8^ The relative frequency (RF) of AOT in Sri Lanka has been reported as 8.6% in 1990^17^ and 4.7% in another study.^17^ Retrospective studies was conducted in Thailand,^18^ China,^19^ Mexico^5^ and California^20^ revealing that the relative frequency of AOT was 5.3% 2.1%, 7.1% and 1.7% respectively.

Two- thirds of the AOTs are diagnosed in the second decade of life and more than half of cases are found in teenagers 13–19 years of age,^2^, ^17^ de Matos et al.^21^ in a retrospective review of 15 cases from Brazil revealed a lower mean age of 16.2 years and studies from California^20^ and China^22^ revealed that the mean ages as 20.2 years and 22.6 years respectively.

The tumor is diagnosed more frequently in women and additional recent studies have revealed a strong female predilection as well.^20^, ^23^, ^24^, ^25^ Further, some other researchers noted that the AOT is more common in blacks.^26^

Although the most common site is anterior maxilla^7^, ^27^, ^28^ there are a few studies which showed a slight mandibular predilection.^29^, ^30^ The predominately associated tooth with AOT wasthe maxillary canine^21^, ^27^ but some studies have revealed a rare involvement of un-erupted molars.^31^ Although AOT is an asymptomatic tumor, patients may be aware of a painless gingival swelling or an area of jaw enlargement which is slowly growing and often associated with an unerupted tooth.^32^

Presence of calcifications gives a mixed radio-dense appearance to AOTs apart from a normal appearance of well- circumscribed unilocular radiolucency.

Histologically it is composed of spindle- shaped epithelial cells that form sheets, strands or whorled masses (rosette like) in a scant fibrous stroma surrounded by a fibrous capsule. Central spaces of the duct- like structures are lined by a layer of columnar or cuboidal epithelial cells that show reversed polarity, suggesting secretory activity. Foci of calcifications may be seen scattered throughout the tumor, and some AOTs contain larger areas of matrix material or calcification which has been interpreted as dentinoid or cementum.^26^ Complete surgical excision with enucleation is the treatment of choice; recurrence of AOT is extremely rare hence very few recurrent cases have been reported.^33^

Several studies have been from different places around the world have been carried out to determine the demographic and clinical profile of AOT according to age, sex, site, extent of tumor and associated impacted teeth. However, in Sri Lanka there are no such studies related to AOT itself. The current study involved 116 cases of AOTs that need to be added to the literature. Therefore, the objective of the present study was to analyze one of the largest series of AOTs from a single center, for a period of thirty eight years, with the existing literature.

## Methods

This study was a retrospective analytical study. The cases which were diagnosed as AOT with their demographic and clinical characteristics (age, sex, location of tumor) from January 1980 − 31st December 2018 were retrieved from the archives of the Department of Oral Pathology, Faculty of Dental sciences, University of Peradeniya, Sri Lanka. AOTs with inadequate data were excluded and cases with multiple biopsies were considered as a single case.

Ethical clearance was obtained from the ethics review committee of the Faculty of Dental sciences, University of Peradeniya. (ERC/FDS/UOP/1/2018/08). Details that are not in the database were retrieved from patients’ request forms which are under Oral Pathology. Histopathologically all cases were evaluated by two pathologists. The cases with unusual features were recorded separately.

Collected data were entered into Microsoft Excel work sheet. Gathered details were grouped according to the age categories to identify the frequency and the 2nd decade group was analyzed separately. Distribution within jaw bones was also evaluated. Data were analyzed using the statistical software SPSS 25 (Statistical Package for Social Sciences 25). Chi-Square test was used to determine the association. Each variable with different combinations were analyzed to identify whether there is any significant relationship. The level of significance was set at (*p* <  0.05) throughout the study.

## Results

A total of 116 cases of AOTs were identified. Age ranged from 5 to 77 years with the mean age of 21.02 years (21.02 ± 11.24) and a median of 18 years. There is a slight difference of the mean ages of females and males (21.14 ± 10.91 years and 20.82 ± 11.91 years).

**Figure 1:**
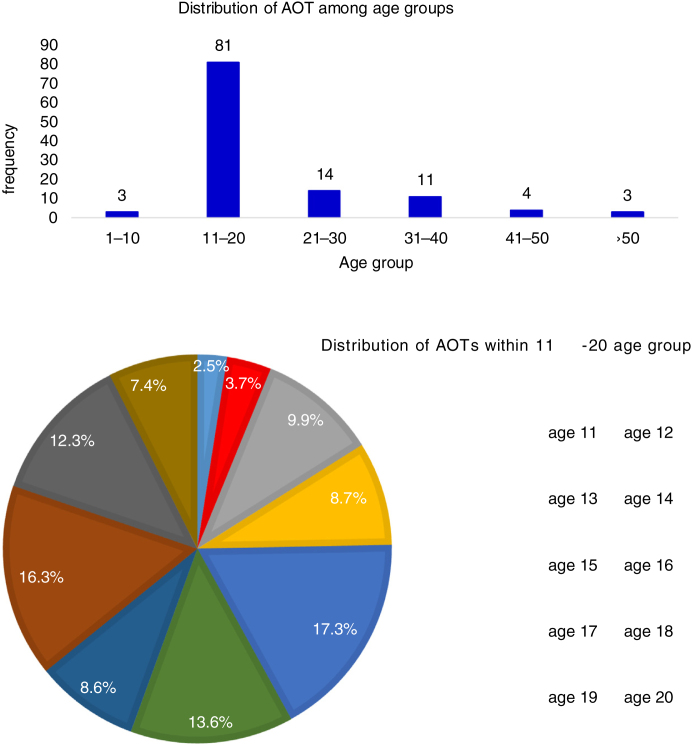
(A) Distribution of AOT among age groups. (B) Distribution of cases within the 11−20 age group.

During the second decade of life the incidence of AOTs is 69.8% which is the highest and followed by 21−30 years age group. From the total sample the peak incidence of AOT (12.1%) was found in the 15 years of age group followed by 18 years of age group (11.2%) and 16 years of age group (9.5%) (Fig. 1a). However, among the 11−20 age group 17.3% were 15 years and 16% of them were 18 years followed by 13.6% who were 16 years old (Fig. 1b).

There were 44 (37.9%) males and 72 (62.1%) females. The male: female ratio for all age groups was 1:1.6. There was a slight female predilection.

**Figure 2:**
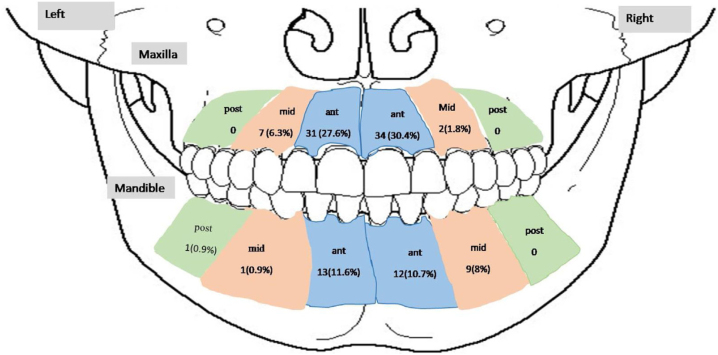
Distribution of AOT cases within jaw bones. Ant, Anterior; Mid, Middle; Post, Posterior.

The maxilla was the commonest site for AOT consisting of 78 cases (67.2%), while 38 (32.8%) cases were reported in the mandible, giving a maxilla-to-mandible ratio of 2.1:1. Out of 116 cases a precise location was identified in 111 cases. The anterior region of both jaws was more frequently affected (81.1%) followed by the middle region (18.0%) and 1 case (0.9%) in the posterior region. In all, 113 cases had information whether the tumour was on right or left. The right side is more frequently affected 60 (53.1%) than the left side 53 (46.9%), giving a ratio of right: left 1.1:1 (Fig. 2). There were two cases (1.8%) in the anterior region without exact site being cited. Therefore, it was not included in Fig. 2.

The maxilla is the site of predilection in the first, second and third decade of life (maxilla: mandible ratio 2:1, 3.5:1 and 1.3:1 respectively). However, findings in the 30 years and over age group cohort, indicated that the mandible was frequently involved. The maxilla: mandible ratio for 31−40 age group was 1:1.75, and for the 41−50 age group was 1:3 and for > 50 years of age, all 3 cases were in the mandible.

The anterior jaw is frequently affected. The ratios for 50 years or above age groups appeared to be, anterior, middle and posterior as 3:0:0, 10.3:1:0, 2:1:0, and 2:0:1 respectively.

According to the analysis of gender distribution and side predilection (left/right), the results indicate the ratio for left side of the jaw for male:female was 1:1.1 and right side of the jaw bone was 1:2.3. Distribution of cases between left and right side of the jawbone is further analyzed according to different decades of life. It shows that right side is the site of predilection in the first, second, third and fourth decade of life (left: right ratio 1:2, 1:1.2, 1:4, and 1:1.5 respectively). However, in the fifth decade of life it shows an equal prevalence for the left and right side of jaw bones. For the patients over 50 years of age, the right side appeared to be the most frequently involved site. Left-to-right ratio for > 50 years age group was 2:1 (Table 1).

**Table 1:** Distribution of AOTs in jaws with gender and age groups.

	Maxilla	Mandible	Total
Male	32	12	44
Female	46	26	72
1−10 years	2	1	3
11−20 years	63	18	81
21−30 years	8	6	14
31−40 years	4	7	11
41−50 years	1	3	4
> 50 years	0	3	3

	Anterior jaw	Middle jaw	Posterior jaw	Total
Male	36	5	1	42
Female	54	15	0	69
1−10 years	3	0	0	3
11−20 years	72	7	0	79
21−30 years	8	4	0	12
31−40 years	4	6	0	10
41−50 years	1	3	0	4
> 50 years	2	0	1	3

	Left	Right	Total
Male	25	18	43
Female	28	42	70
1−10 years	1	2	3
11−20 years	39	42	81
21−30 years	5	7	12

There was a statistically significant relationship with site of occurrence (maxilla/mandible) and age (*p* < 0.005). Further, depending on whether it occurs in anterior/mid/posterior also showed a significant relationship with age (*p* ≤  0.001). However, side of occurrence (left/right) with age and site of occurrence with gender were not significant statistically

**Figure 3:**
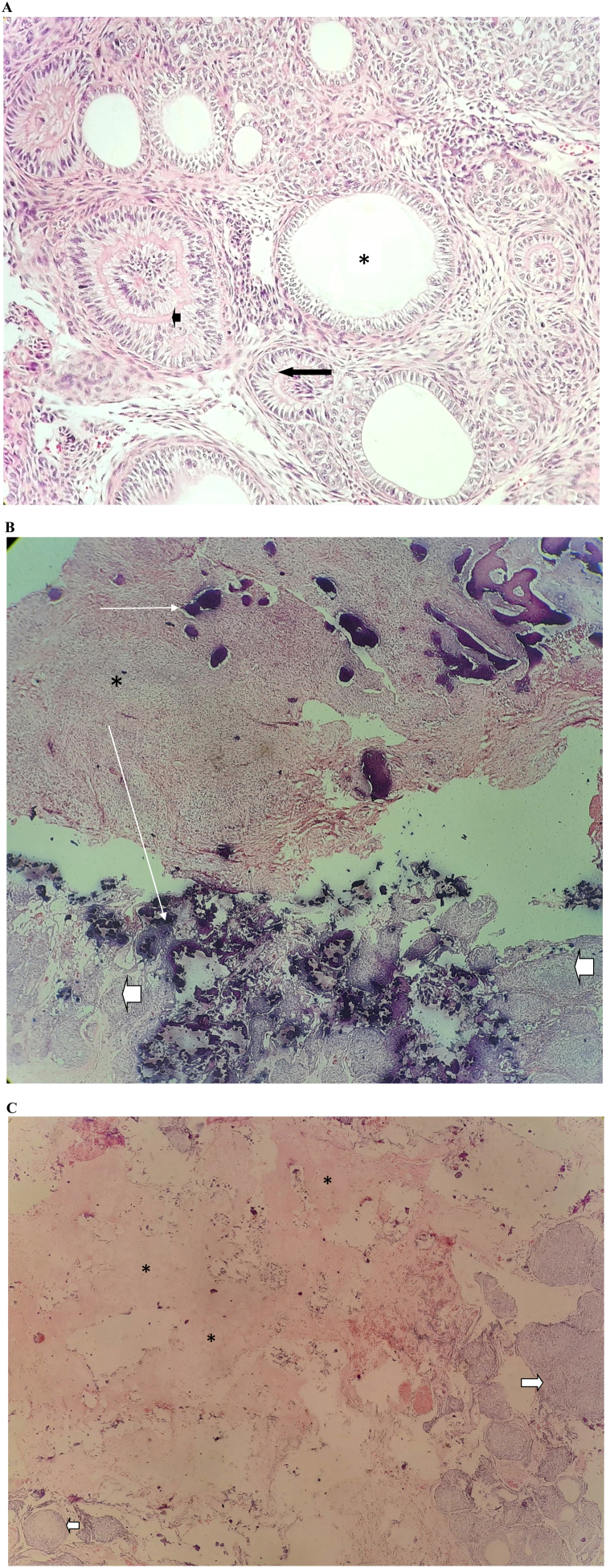
(A) Histological appearance of AOT. (B) AOT with numerous calcifications. (C) AOT with eosinophilic material mimicking dentin (asterisk) higher magnification.

The majority of the cases showed characteristic features of AOTs (Fig. 3a) with one case presenting as a large mandiblular lesion. Some cases showed remarkable calcifications while others showed homogenous eosinophilic material (Fig. 3b−c).

## Discussion

The adenomatoid odontogenic tumor is a benign odontogenic lesion that has been regarded either as a non-invasive, non-aggressive neoplasm or as a developmental hamartomatous growth.^26^, ^34^ Several studies from different places around the world have been carried out to determine the demographic and clinical profile of AOT. According to previous studies the demographic and clinical presentation of AOT does not differ significantly from one country to other.^2^, ^17^ However, in Sri Lanka there is no updated information available regarding demographic and clinical profile of AOT. Therefore, this study was undertaken to analyze demographic characteristics and clinical profile of AOTs in Sri Lanka.

Adenomatoid odontogenic tumor is not a common OT. Therefore, we have only 116 cases on record at the Department of Oral Pathology, Faculty of Dental sciences, University of Peradeniya for the past 38 years. However, this is the largest sample from a single institution so far in the literature. The result of our study is in par with most studies around the world.

It had been generally accepted that the relative frequency of AOT corresponds to 2.2%−8.7% of all odontogenic tumours.^8^, ^31^ However, in a worldwide collaborative retrospective study, the relative frequency of AOT ranged from 0.6% to 38.5%.^34^ The relative frequency of AOT in Sri Lanka reported as 8.6% in 1990.^17^ However, more recent studies from Sri Lanka reported a lower relative frequency, which was 4.7% of all OTs.^2^ Likewise, some reports from China suggested a higher relative frequency (8.3%) of AOT.^22^ Although recent retrospective review of 1309 cases from China revealed a lower relative frequency as 2.1%.^19^ In comparison with Asian countries, our relative frequency was lower than those reported in countries such as Thailand (5.3%).^18^ Studies from Malaysia (0.3%) China (2.1%)^19^, ^29^ California,^20^ Nigeria,^25^ Brazil,^21^ and Mexico^5^ found the relative occurrence of AOT among total OTs, as 0.3%, 2.1%, 1.7%, 4.5%, 5.4% and 7.1% respectively.

Similar to the present study, retrospective studies with large case series revealed a female predominance for AOT, with global female-to-male ratio of 1.9:1.^8^, ^34^ However, the female-to-male ratio of 1.6:1 obtained on this study did not reflect the marked female preponderance with previous studies in Asia. A recent study from Sri Lanka revealed female-to-male ratio as 2:1.^2^ Toida et al.^35^ in Japan have reported that the female-to-male ratio of 3.0:1. In contrast, Swasdison et al.^18^ in a retrospective review of 67 cases for Thai population showed female-to-male ratio as 1.8:1 which was closely similar with our findings. Furthermore, Arotiba et al.^27^ in a previous study from Nigeria and de Matos et al.^21^ in a review of 15 cases from Brazil showed a female-to-male ratio of 1.4:1, which was more in line with our findings.

With respect to age distribution, it has been reported that more than two-thirds of AOTs are diagnosed in young patient, especially those in the second decade of life and more than 80% are found before the age of 30 years.^8^, ^27^, ^34^ Our study findings are in agreement with these reports.

The mean age of the patient at the time of diagnosis was 21.02 years. It was 21.14 years for females and 20.82 years of mean age for males in the current study. Our previous studies showed a lower mean age probably due to a lesser number of cases compared with the present study (17.6 years and 18 years respectively).^2^, ^17^

However, Swasdison et al.^18^ in a retrospective review of 67 cases from Thailand indicated that 21.1 yearsis the mean age: 21.4 years as a mean age for males and 20.9 years as a mean age for females. Current results were more in parallel with this study. Mean ages from studies by Adisa et al.^25^ from Nigeria and Lu et al.^22^ from China were 20.4 years and 22.6 years respectively. In addition a study composed of 1088 cases of OTs from Northern California reported 20.2 years as the mean age.^20^ The findings of the above studies were more in keeping with the findings of the current study. Furthermore, Ochsenius et al.^7^ in a retrospective review from Chile revealed a similar mean age (21.03 years) compared to current study findings. Leon et al.^36^ in a clinicopathological and immunohistochemical study of 39 cases of AOT from three oral diagnostic services (Brazil, Mexico and Guatemala) and de Matos et al.^21^ in a retrospective review of 15 cases from Brazil showed lower mean ages of 16 years and 16.2 years respectively. Arotiba et al.^27^ in a retrospective review of 57 cases for Black African population reported the mean age as 17 years. However, current study findings are not compatible with these findings.

AOT occurs predominantly in the maxilla (67.2%) compared to mandible (32.8%), with maxilla-to-mandible ratio of 2.1:1 (showing female-to male ratio of 1.4:1 for maxilla and 2.1:1 for mandible), anterior part of the jaw (81.1%) were much more affected than the mid- (18.0%) and posterior regions of the jaw (0,9%). Furthermore, the right side (53.1%) was affected slightly more compared with the left side (46.9%).The maxilla-to-mandible ratio of AOT for Sri Lanka previously reported as 2.3:1, in parallel with the findings of the current study.^2^ Similar studies carried out worldwide revealed a maxillary predilection with maxilla-to-mandible ratios of 1.25:1 (25), 1.8:1 (36), 1.9:1 (18) and 2:1.^31^ The predilection for the anterior region of the jaw was revealed in some studies.^18^, ^25^ In addition to a retrospective review of 15 cases from Brazil, a similar study from Chile also revealed an anterior jaw predilection.^7^, ^21^ Current study results were more in line with those studies.

Although, retrospective studies from Malaysia and Brazil showed a slight mandibular predilection,^29^, ^30^ our results and most other studies contradict the above findings. Some older studies from Nigeria suggested a mandibular predilection for AOT.^37^ However more recent studies from Nigeria by Arotiba et al.^27^ and Effiom et al.^28^ agree with an anterior maxillary preponderance.

## Conclusion

The results from the present study observed that AOT occurs more frequently in the second decade of life, more prevalent in females, most often associated with maxilla, predominantly affecting anterior jaw bones and presenting mostly in the right side. The results from the present study showed the statistically significant relationship with site of occurrence (maxilla/mandible) and age (*p* < 0.005). Further, depending on whether it occurs in anterior/mid/posterior also showed a significant relationship with age (*p* ≤ 0.001). However, side of occurrence (left/right) with age (*p* > 0.05) was not statistically significant and there was no statistically significant relationship with site of occurrence with age. In addition, this study revealed a few differences on demographic and clinical presentation of AOT from region to region.

## Data availability statement

Data used for analysis can be produced whenever requested. Raw data also can be provided with request.

## Conflicts of interest

The authors declare no conflicts of interest.
